# *Kif3a* deletion from posterior limb regions results in ectopic anterior digits in mice.

**DOI:** 10.17912/micropub.biology.002438

**Published:** 2026-10-26

**Authors:** Natalia Shylo, Stephanie Kacik, Scott D. Weatherbee

**Affiliations:** 1 Biological and Biomedical Sciences Department, Rowan University, Glassboro, NJ, USA; 2 Department of Genetics, Yale University, New Haven, CT 06520, USA; 3 Bone Marrow Transplant and Cellular Therapy Program, Yale New Haven Hospital, New Haven, CT, USA; 4 Biology Department, Fairfield University, Fairfield, CT 06824, USA.

## Abstract

Modern tetrapod limbs display diverse morphologies from bat wings to cow hooves and beyond. Sonic hedgehog (Shh) signaling has an evolutionarily conserved role in specifying the number and shape of posterior digits and requires functional cilia to do so. To test if posterior limb cilia are required for Shh-mediated digit specification, we disrupted *Kif3a* , a critical motor protein essential for cilia formation, in mouse posterior forelimb mesoderm, using the *Hoxb6-Cre* transgenic line. Strikingly, we observed ectopic anterior digits in *Hoxb6-Cre;Kif3a ^Flox/Δ^* forelimbs. These ectopic anterior digits originated from posterior limb cells and were independent of ectopic Shh signaling.

**Figure 1. Kif3a deletion from the entire hindlimb mesoderm or posterior forelimb mesoderm results in ectopic anterior and posterior digit formation and Shh patterning defects:** A-D, alcian blue (cartilage) and alizarin red (bone) staining of E18.5 mouse forelimbs (A, B) and hindlimbs (C, D). Wildtype forelimbs (A) develop 5 digits (1-5) with 3 phalanges in each of the posterior digits (2-5) located distal to the metacarpal (mc), whereas anterior digit 1 only develops 2 phalanges (n=18 forelimbs). Wildtype hindlimbs (C) display similar digit patterns (n=18 hindlimbs). *Hoxb6-Cre;Kif3a ^Flox/Δ^* forelimbs develop 6-8 digits (B; 6 digits, n=14; 7digits, n=11; 8 digits, n=5), including ectopic digits on both the anterior (asterisk) and posterior (bracket) sides of the limb. *Hoxb6-Cre;Kif3a ^Flox/Δ^* anterior forelimb digits have 2 phalanges, whereas posterior digits have gaps between the metacarpal elements and distal phalanges. *Hoxb6-Cre;Kif3a ^Flox/Δ^* hindlimbs form 6-8 digits (D; (6 digits, n=12; 7 digits, n=15; 8 digits, n=3)) that are similar in morphology and, like the posterior forelimb digits, display gaps between the metatarsal elements and distal phalanges. E-P, E11.5 limb RNA *in situ* hybridizations detecting *Shh* (E-H), *Gli1* (I-L), or *Grem1* (M-P) transcripts, in purple. At E11.5, *Shh* expression is normally restricted to the posterior region of the limbs (E,G; n=8 limbs) and is found in a similar region in *Hoxb6-Cre;Kif3a ^Flox/Δ^* limbs (F,H; n=8 limbs). *Gli1* is expressed in the posterior ~2/3 of control limbs (I,K; n=8 limbs) at E11.5 but is absent from *Hoxb6-Cre;Kif3a ^Flox/Δ^* limbs (J,L; n=8 limbs). At E11.5, *Grem1* is expressed in a posterior-biased arc in wildtype limbs (M, O; n=8 limbs). *Hoxb6-Cre;Kif3a ^Flox/Δ^* forelimbs develop ectopic anterior patches of *Grem1* expression (N, asterisk; n=4 forelimbs) while hindlimbs show a continuous expansion of *Grem1* expression to the anterior edge of the limb (P, bracket; n=4 hindlimbs). Q-T, E13.5 and U-X, E18.5 *Hoxb6-Cre;Rosa-EYFP* (control; Q,R,U,V) and *Hoxb6-Cre;Kif3a ^Flox/Δ^* (S,T,W,X) forelimbs under brightfield (Q,S,U,W) or fluorescent (Rosa-EYFP; R,T,V,X) illumination. *Rosa-EYFP* expression in control forelimbs shows that descendants of *Hoxb6-Cre* -expressing cells contribute to digits 2-5 (R, V; n=11 embryos), but do not populate digit 1. In contrast, in *Hoxb6-Cre;Kif3a ^Flox/Δ^* forelimbs, *Hoxb6-Cre* descendants are found in the anteriormost, ectopic digits in *Hoxb6-Cre;Kif3a ^Flox/ΔD^* forelimbs (T, X, asterisks; n=8 embryos). All scale bars = 0.5 mm.

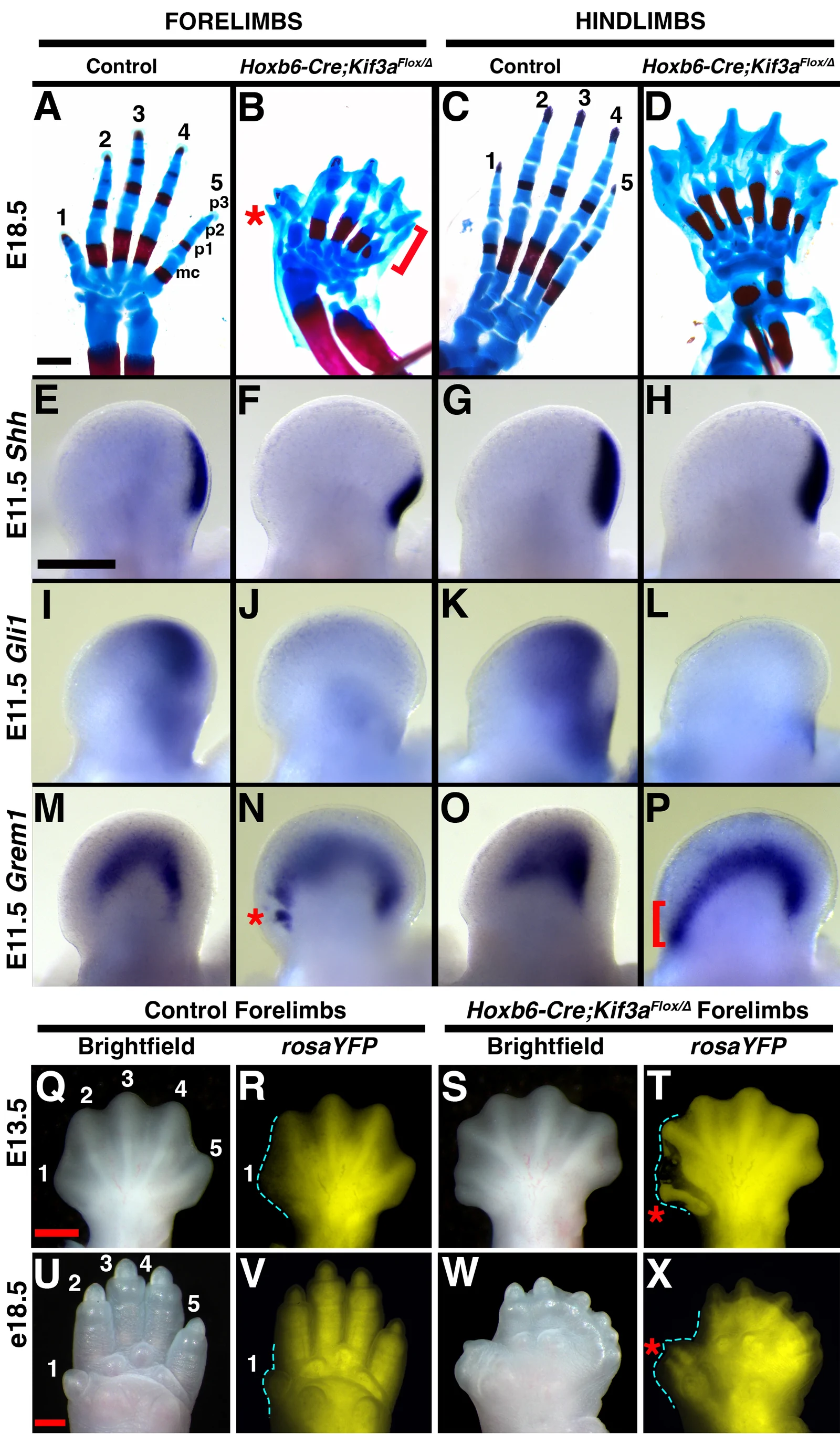



## Description

A common feature of tetrapod limbs is the critical role of Shh signaling in specifying digit number and identity (Tickle & Towers, 2017)). In mice lacking functional Shh, a single digit with anterior (digit 1) identity forms (Chiang et al., 1996). In contrast, application of Shh protein or activation of the Shh pathway in anterior limb regions induces ectopic digit formation and transformation of anterior digits to more posterior identity (Riddle et al., 1993; Yang et al., 1997). Additional complexity in Shh signaling comes from a dependence on cilia for transduction of the pathway (Huangfu et al., 2003) and several studies have shown that mice with mutations in genes that affect cilia formation or function develop limbs with ectopic digits (Christopher et al., 2012; Haycraft et al., 2005; Liu et al., 2005; Weatherbee et al., 2009; Xin et al., 2017). We set out to disrupt cilia in posterior limb mesoderm to determine if there was a posterior-specific requirement for cilia given the posterior restriction of Shh activity in limbs. To achieve this, we combined a conditional knockout allele of *Kif3a* (Lin et al., 2003) with the *Hoxb6-Cre* (Lowe et al., 2000) transgenic line, which drives Cre recombinase expression in a posteriorly restricted manner in forelimbs, in the precursors of digits 2-5 (Lowe et al., 2000; Nguyen et al., 2009; Zhu et al., 2008). If Shh signaling in limbs acts only through posterior cilia, we expected to observe posteriorly-restricted digit patterning defects in *Hoxb6-Cre;Kif3a ^Flox/Δ^* forelimbs.

*Hoxb6-Cre;Kif3a ^Flox/Δ^* mouse embryos form ectopic digits

Wildtype mouse forelimbs form five digits, with digit 1 consisting of 2 phalanges and the posterior digits comprising 3 phalanges each ( Figure 1A ). In all wildtype digits, the proximal phalange (p1) abuts the respective metacarpal (mc). At E18.5, *Hoxb6-Cre;Kif3a ^Flox/Δ^* forelimbs show 6 or more digit tips, including a duplicated or partially duplicated digit 1 ( Figure 1B, asterisk) as well as additional posterior digits. Posterior *Hoxb6-Cre;Kif3a ^Flox/Δ^* digits also show a loss of proximal phalanges resulting in a gap between metacarpals and phalanges ( Figure 1B, bracket). Duplications of digit 1 are particularly notable in *Hoxb6-Cre;Kif3a ^Flox/Δ^* forelimbs as there is no reported *Hoxb6-Cre* recombinase activity in digit 1 precursor mesoderm (Lowe et al., 2000).

Wildtype hindlimbs are patterned similarly to forelimbs ( Figure 1C ). *Hoxb6-Cre* is expressed throughout hindlimb mesoderm (Lowe et al., 2000) and *Hoxb6-Cre;Kif3a ^Flox/Δ^* hindlimbs develop 6 or more digits that are uniform in appearance, lacking anterior/posterior digit identities, each consisting of a single distal phalange separated from the corresponding metatarsal by a gap where the proximal phalanges should form ( Figure 1D ). This phenotype is distinct from the forelimb defects, where anterior digit identities (2 phalanges) are partially maintained, and instead more closely resembles previously-reported conditional deletion of Ift88 or Kif3a using *Prx1-cre* (Haycraft et al., 2007). Note that proximal limb elements were also shortened in *Hoxb6-Cre;Kif3a ^Flox/Δ^* limbs but their characterization is beyond the scope of this study.

Shh signaling is disrupted in *Hoxb6-Cre;Kif3a ^Flox/Δ^* limb buds

To test whether the abnormal digit morphologies and ectopic digits observed at E18.5 in *Hoxb6-Cre;Kif3a ^Flox/Δ^* limbs stem from disruptions in early Shh signaling, we examined the expression of Shh and two downstream target genes in E11.5 limbs. Posterior-restricted expression of *Shh* was observed in *Hoxb6-Cre;Kif3a ^Flox/Δ^* limbs (Figure1 F,H) similar to control limbs (Figure1 E,G) at E11.5. This indicates that the ectopic forelimb digits in *Hoxb6-Cre;Kif3a ^Flox/Δ^* animals are not the result of ectopic *Shh* expression.

The Gli transcription factors (Gli1,2,3) are expressed in limbs and act as transcriptional activators (GliA) in response to Shh ligand in the posterior limb field and function as ligand-independent transcriptional repressors (GliR) of Shh targets in the anterior limb (Litingtung et al., 2002; te Welscher et al., 2002; C. Wang et al., 2007). Gli1 acts solely as an activator (Dai et al., 1999; Park et al., 2000; Sasaki et al., 1997), while Gli2 and Gli3 acts as both activators (GliA) and repressors (GliR) in the limb (Bowers et al., 2012; Pan et al., 2009; B. Wang et al., 2000). Cilia are required for processing of Gli proteins into functional GliA and GliR forms and mutations in genes that affect cilia formation or function affect both GliA and GliR functions (Haycraft et al., 2005; Liu et al., 2005). Hedgehog signaling leads to GliA activation that directly induces *Gli1* , which further acts as an additional source of GliA signaling (Bai & Joyner, 2001). We used *Gli1* expression as a readout of GliA function in *Hoxb6-Cre;Kif3a ^Flox/Δ^* limbs. In wildtype E11.5 embryos *Gli1* is expressed in the posterior 2/3rds of fore- and hindlimb buds (Figure1 I,K). In *Hoxb6-Cre;Kif3a ^Flox/Δ^* limbs, *Gli1* expression is absent from both fore- and hindlimbs (Figure1 J,L) indicating a loss of GliA activity throughout the limb following *Kif3a* deletion. Therefore, the ectopic forelimb digits in *Hoxb6-Cre;Kif3a ^Flox/Δ^* animals are not the result of increased or ectopic GliA actvity.

*Gremlin 1* ( *Grem1* ) expression is induced by GliA in the posterior limb bud and is also repressed by GliR activity in the anterior limb bud (Lewandowski et al., 2015; te Welscher et al., 2002; Vokes et al., 2008) resulting in a posterior-biased arc of *Grem1* expression (Figure1 M,O). In *Hoxb6-Cre;Kif3a ^Flox/Δ^* hindlimbs, *Grem1* expression expands to the most anterior region of the limb (Figure1P, bracket). The absence of expression of the GliA target, *Gli1,* in *Hoxb6-Cre;Kif3a ^Flox/Δ^* limbs rules out the possibility that ectopic GliA activity in anterior regions underlies anterior *Grem1* expansion. Instead, anteriorly expanded *Grem1* expression is most likely due to the loss of inhibitory GliR activity by Gli2/3 in anterior *Hoxb6-Cre;Kif3a ^Flox/Δ^* limb tissue.

The loss of *Gli1* expression and anterior expansion of *Grem1* expression in *Hoxb6-Cre;Kif3a ^Flox/Δ^* hindlimbs are both consistent with previously reported conditional deletion of *Kif3a* using *Prx1-cre* (Haycraft et al., 2007), indicating a complete loss of cilia function in *Hoxb6-Cre;Kif3a ^Flox/Δ^* hindlimbs. In contrast, *Hoxb6-Cre;Kif3a ^Flox/Δ^* forelimbs show ectopic anterior patches of *Grem1* expression, but they do not extend to the anterior edge of the limb ( Figure 1N, asterisk). The ectopic *Grem1* expression in anterior forelimb regions was surprising as *Hoxb6-Cre* activity is reportedly absent from the anterior forelimb (Lowe et al., 2000; Nguyen et al., 2009). This suggested the possibility that a subset of anterior forelimb cells had lost GliR activity in *Hoxb6-Cre;Kif3a ^Flox/Δ^* embryos.

*Hoxb6-Cre;Kif3a ^Flox/Δ^* descendants contribute to anterior forelimb digits

The duplication of digit 1 and patchy *Grem1* expression in *Hoxb6-Cre;Kif3a ^Flox/Δ^* forelimbs led us to examine where Cre recombinase was active (and thus where *Kif3a* was deleted) in *Hoxb6-Cre;Kif3a ^Flox/Δ^* forelimbs by crossing in the *Rosa-EYFP* reporter. The *Rosa-EYFP* reporter line consists of a stop sequence flanked by loxP sites, located upstream of an enhanced yellow fluorescent protein gene. When crossed with a Cre-recombinase line, EYFP expression occurs in cells that express Cre-recombinase as well as their descendants (Srinivas et al., 2001). In control *Hoxb6-Cre; Rosa-EYFP* embryos, *EYFP* expression was restricted to forelimb digits 2-5, and absent from digit 1 ( Figure 1R, V,) similar to previous studies using *Hoxb6-Cre;Rosa-LacZ* (Nguyen et al., 2009). In contrast, in *Hoxb6-Cre;Kif3a ^Flox/Δ^ ; Rosa-EYFP* embryos, *Rosa-EYFP* expression was not excluded from anterior forelimb regions. Early digit formation stages show EYFP+ (Kif3a-deleted) cells in anterior digits, often in proximal-distal streams with distinct boundaries ( Figure 1T, asterisk). At E18.5, EYFP+ cells were consistently found in anterior digits in *Hoxb6-Cre;Kif3a ^Flox/Δ^ ; Rosa-EYFP* forelimbs ( Figure 1X, asterisk). This suggests that disrupting cilia in the posterior forelimb causes cells that originated in the posterior to give rise to ectopic anterior digits.

Our initial hypothesis for the posterior restriction of Shh activity in vertebrate limbs was that cilia restrict the diffusion of Shh across the limb field. Strikingly, deletion of a critical cilia gene (Kif3a) from the posterior forelimb mesoderm results in ectopic digits on the anterior side of the limbs ( Figure 1A, B). However, our data indicate that the ectopic anterior digits are not due to ectopic *Shh* expression or abnormal activation of GliA targets in the anterior limb. Instead, by using a lineage tracer, we observed that cells with functional Kif3a ( *EYFP-* ) and Kif3a-deleted ( *EYFP+* ) cells seem to segregate from each other ( Figure 1T ) but can change anteroposterior positions allowing for more posterior-originating cells ( *EYFP+* ) to end up in anterior digits. This cell rearrangement could explain the ectopic anterior patches of *Grem1* expression in *Hoxb6-Cre;Kif3a ^Flox/Δ^* forelimbs ( Figure 1N ) possibly through the loss of GliR activity, although the mechanism underlying the rearrangement will require additional studies.

## Methods

**Mouse Lines :**

**Table:** 

**MOUSE STRAIN**	**SOURCE**
* Kif3a ^Flox^*	Stefan Somlo, Yale University (Lin et al., 2003)
* Kif3a ^Δ^*	Stefan Somlo, Yale University (Lin et al., 2003)
* Rosa-EYFP (Gt(ROSA)26Sor ^tm1(EYFP)Cos^ ) *	JAX: The Jackson Laboratory
*Hoxb6-Cre (Tg(Hoxb6-cre)#Mku)*	JAX: The Jackson Laboratory

All mice were maintained on a mixed genetic background. The *Hoxb6-Cre* (Lowe et al., 2000), *Kif3a ^Flox/+^* (Lin et al., 2003), *Kif3a ^Δ/+^* (Marszalek et al., 1999), and *Rosa-EYFP* (Srinivas et al., 2001) mouse lines and genotyping have previously been described. To facilitate deletion of *Kif3a* by *Hoxb6-Cre* , we crossed heterozygous carriers of the conditional *Kif3a* allele ( *Kif3a ^Flox/+^* ) and the null allele ( *Kif3a ^Δ/+^* ) to generate *Kif3a ^Flox/Δ^* mice, which presented with a wildtype phenotype. Mouse experiments were performed in accordance with Yale Institutional Animal Care and Use Committee guidelines. For time-staged embryos, E0.5 was assigned to noon of the day the vaginal plug was detected.

**Skeletal staining**

Skeletons were prepared and stained with Alcian Blue to mark cartilage and Alizarin Red to visualize bone, using standard methods (Behringer et al., 2014), as described previously (Xin et al., 2026).

**RNA Riboprobes :**

**Table:** 

**GENE**	**VECTOR**	**SOURCE**
*Gli1*	pBluescript	A. Joyner (Hui et al., 1994)
*Grem1*	pBluescript	R. Harland (Khokha et al., 2003)
*Shh*	pBluescript II	A. McMahon (Zúñiga et al., 1999)

The *in situ* hybridization protocol was performed using standard methods (Behringer et al., 2014), as previously described (Christopher et al., 2012).
